# Organization of the Medical Profession of Georgia

**Published:** 1886-09

**Authors:** 


### ORGANIZATION OF THE MEDICAL PROFESSION OF GEORGIA.

A movement is on foot for the thorough organization of the Medical Profession of Georgia. The effort is a commendable one, and should meet the hearty approval and earnest aid of every regular physician in the State, and it is to be hoped that it will, for the interests of the profession and of the people demand that it should.

This movement was inaugurated by the very concise and comprehensive report which Dr. Wm. Abram Love, of Atlanta, made to the State Medical Association, at Augusta, on the working of the Medical Association of the State of Alabama, he having been appointed a special delegate to that body.

His report is replete with interest, and we regret that we cannot present it in full in the present number of THE JOURNAL. It will appear in a forthcoming issue. Those who feel an interest in the movement should study that report closely. It shows clearly what can be accomplished by organization and earnest, united work. The Secretary of the Alabama Association, Dr. Means, having received and carefully examined a copy, writes of it: "This report is exceedingly creditable to Dr. Love, as it embodies in concise form the working of our model Association. It should be sent to every medical organization in the States of this Union, being, as it is, by far the most complete explanation yet given of our system of work and the beautiful machinery by which such work is accomplished. In the next volume of our Transactions, the report will appear in full."

Such is the reception given the report in Alabama. By the Medical Association of Georgia it was received with a vote of thanks and ordered printed.

Under resolutions of the Association a committee was appointed to open correspondence with some of the leading working members of the profession in each county, urging upon them the organization, by counties, into county medical societies, and the appointment of delegates to the next annual meeting of the State Association.

We earnestly urge it upon the members of the profession throughout the State to second this movement. Want of united, concerted action, for want of proper organization, has been a curse to the profession of Georgia. Why should such a condition of things longer exist? Let the regular physicians of every county, respectively, organize and send delegates to the next annual meeting of the Association. There was never a better time to remedy the evil and elevate our standard than now. It can be done, it must be done, and it will be done!

Any member of the profession who will give aid or advancement to this movement, or may desire further information regarding the same, would do well to confer directly with Dr. Love, who was appointed chairman of the committee and who, having the matter in charge, will prove a zealous worker in effecting a thorough organization.

